# Magnetized radiative flow of propylene glycol with carbon nanotubes and activation energy

**DOI:** 10.1038/s41598-023-49150-w

**Published:** 2023-12-09

**Authors:** Hassan Ali Ghazwani, Muzher Saleem, Fazal Haq

**Affiliations:** 1 Department of Mechanical Engineering, Faculty of Engineering, Jazan University, P.O Box 45124, Jazan, Kingdom of Saudi Arabia; 2 Department of Mathematical Sciences, Karakoram International University Main Campus, Gilgit, 15100 Pakistan

**Keywords:** Mathematics and computing, Nanoscience and technology

## Abstract

Carbon nanotubes (CNTs) have gained significant attention in the field of fluid dynamics and fluid flows due to their unique properties and the potential to enhance various aspects of fluid transport features. This article explores the behavior of Darcy–Forchheimer flow of Propylene glycol $\left( {C_{3} H_{8} O_{2} } \right)$ submerged single wall carbon nanotubes (SWCNT) and multi-wall carbon nanotubes (MWCNT). The flow features are examined over stretched preamble surface of sheet. Energy relation is acquired in manifestation of thermal radiation and Joule heating. Aspects of Arrhenius kinetics and chemical reaction are assimilated in mass transport relation. Furthermore, effects of intermolecular fluid friction is accounted. Flow prevailing mathematical model is acquired by implementing boundary layer assumptions. Transformations procedure is adapted to alter the dimensional model into non-dimensional one and then tackled through Runge–Kutta–Fehlberg method (RKF-45) in Mathematica. Effective consequences of influential flow controlling parameters on fluid velocity, thermal transport and concentration are inspected by plotting. Numerical computations for interesting engineering quantities like skin friction coefficient, mass and heat transfer rates are tabulated and investigated. It is noticed that thermal field boosts versus curvature variable, Eckert and Hartmann numbers. Retardation in mass concentration is noticed via Schmidt number and activation energy variable. Velocity field shows decreasing trend through porosity parameter, Hartmann number and Darcy–Forchheimer variable. Furthermore, it is noticed that magnitude of skin friction coefficient is higher for SWCNT as compared to MWCNT.

## Introduction

Carbon nanotubes are cylindrical nanostructures composed of carbon atoms arranged in a unique hexagonal lattice. They have exceptional mechanical, electrical, and thermal properties, making them valuable in various applications ranging from electronics and aerospace to medicine and materials science. CNTs can be classified into two main types: SWCNT and MWCNT. Both SWCNT and MWCNT have unique properties and applications. CNTs first time introduced by Iijima^1^ in 1991 because of their unique features. Suhr et al.^2^ studied the features of viscoelasticity in composites of CNTs. Tribological features of CNTs strengthened by copper mixtures were inspected with dry conditions by Tu el al.^3^. An innovative model for effective thermal conductivity considering CNTs based on Maxwell theory is proposed by Xue^4^. Ramesh and Madhukesh^5^ extended the notion of concentration and inspected the aspects of chemical reaction and Arrhenius’s kinetics in flow of hybrid CNTs accounting the features of induced magnetic field. Hayat et al.^6^) presented flow of CNTs based fluid saturated in a rotating frame with Darcy–Forchheimer topographies. Influence of inclined magnetic field and thermal radiation in Casson fluid immersed SWCNT and MWCNT flow caused by absorptive dwindling sheet is reported by Mahesh et al.^7^. Anusha et al.^8^ scrutinized the heat transport features in radiative Jeffrey fluid immersed CNTs as nanoparticles with Navier’s slip and MHD effects. Theoretical exploration of radiated Maxwell nanofluid flow between stretchable disks containing CNTs with convective boundary restrictions is reported by Reddy et al.^9^. Diverse features of CNTs in flow of nanofluid are explored by Raja et al.^10,11^.

Magnetohydrodynamics (MHD) is a multidisciplinary field that combines principles from both magnetism and fluid dynamics to study the performance of electrically conducting liquids, such as plasmas, liquid metals and ionized gases in the presence of magnetic fields. It is often used to describe and analyze the behavior of materials like plasma in fusion reactors, the solar wind, the Earth's core and other astrophysical and engineering applications where the interaction between magnetic fields and conductive fluids is important. It has applications ranging from astrophysics to nuclear fusion research and continues to be an active area of scientific investigation and engineering development. Features of MHD flow of water based nanofluid immersed nano sized particles of copper in an inflexible circular disk is reported by Abdulaziz and Alkuhayli^12^. Alzabut et al.^13^ considered mixed convection MHD flow of Newtonian fluid in a differentially animated rectangular enclosure. Transport features of MHD flow of viscous fluid considering the influences of first order reaction, inclined magnetic field and thermal radiation is examined by Sadighi et al.^14^. Simultaneous impacts of magnetic field and chemical reaction in forced convective radiated Cross nanofluid flow is reported with the help of artificial neural network by Jakeer et al.^15^. Rasool et al.^16^ scrutinized numerically the inspirations of chemical reaction and viscous dissipation in Williamson nanofluid flow by stretched sheet. Axisymmetric flow of MHD third grade liquid beside an elongating cylinder is reported by Hayat et al.^17^. Consequences of bioconvection phenomenon in MHD thixotropic nanomaterial flow is probed by Shafiq et al.^18^. Consequences of thermal radiation and Arrhenius kinetics in MHD flow of Cross liquid by the surface of stretched parabola is inspected by Awais and Salahuddin^19^.

Now a days the researchers are interested in investigating the flow of fluids through porous medium because of their various practical applications in agricultural field, engineering, petroleum technology, fluid mechanics, process of mineral and mining and production of oil and gas. Accessible literature certifies that Darcy’s theory has been frequently applied for modeling and investigation of flow related problems. It is observed that this theory is good for less porosity and lower velocity. Therefore, this theory lacking to explain the behavior when boundary effects and inertia occur at higher rate of flow. Forchheimer^20^ address the deficiency by adding velocity square term in momentum relation. Muskat^21^ designated this extension factor as Darcy–Forchheimer (DF). Seddeek^22^ illustrated mixed convection flow in light of Darcy–Forchheimer relation. DF flow of hydromagnetic nanomaterial by a stretchable porous surface with Ohmic dissipations and heat source/sink is inspected by Ganesh et al.^23^. Jawad et al.^24^ surveyed the influence of variable thermal conductivity in DF flow of Maxwell fluid with constraints of convective and zero mass flux type. Ullah et al.^25^ reported the influence of absorption/generation and slips in DF flow triggered by a rotating disk. Çolak et al.^26^ examined the behavior of bioconvective DF flow of Powell Eyring fluid with slip and convective boundary restrictions. Mass, heat and motile density transfer rates in mixed convection DF flow of nanoliquid is scrutinized through improved Fourier and Fick’s laws by Raja et al.^27^. Awais et al.^28^ inspected the characteristics of radiated DF flow of Eyring-Powell fluid with Dufour and Soret features. Upreti et al.^29^ scrutinized the behavior of water based DF flow immersed CNTs. References^30–34^ explores various aspects of DF flow of nanofluids by stretchable surfaces.

The aforementioned studies highlights various aspects of nanofluid flows caused by stretching and rotating geometries. Nevertheless, to the best of author’s understanding, the DF flow of magnetized Propylene glycol immersed SWCNT and MWCNT as nanoparticles by the stretched surface is not yet examined. So the main objective of present work is to examine the influences of binary chemical reaction, Arrhenius kinetics and viscous dissipation in DF flow of magnetized Propylene glycol immersed SWCNT and MWCNT as nanoparticles. The flow governing equations are developed considering the flow over the surface of permeable cylinder. The model equations representing the flow are solved via RKF-45 scheme. A brief graphical and numerical explanation is highlighted.

## Problem structure

**Figure 1:**
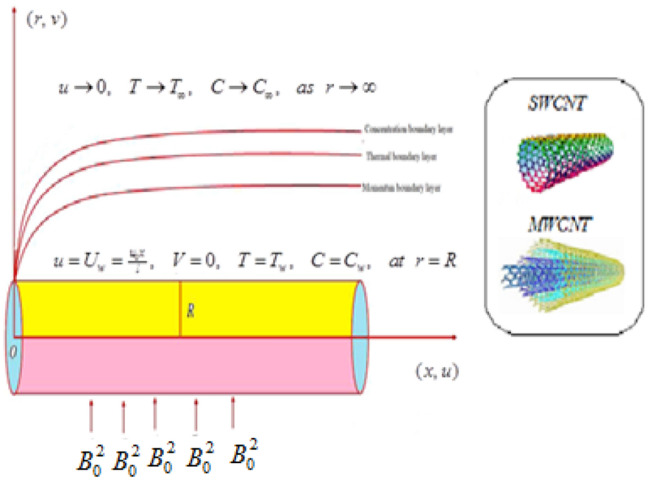
Flow geometry.

Here, flow of hybrid nanofluid propylene glycol submerged SWCNT and MWCNT as nanoparticles is investigated. The flow characteristics are examined by stretched cylinder having porous walls. The effects of Darcy–Forchheimer, thermal radiation, Joule heating and inter molecular friction force are considered in modeling. Furthermore, chemical reaction associated with Arrhenius kinetics is executed at the surface of cylinder. The cylinder is supposed to be stretched along $x -$ direction with velocity $u_{w} \left( { = \tfrac{{u_{0} x}}{l}} \right)$. $T_{w}$ and $T_{\infty }$ be the surface and ambient temperatures of fluid respectively. A uniform magnetic field is imposed perpendicular to the flow. The schematic flow diagram is depicted in Fig. 1. Tensor equations for mass conservation, momentum, energy and concentration are as follows^5,35,36^;

1 $$\nabla .\mathop V\limits^{ \to } = 0,$$

2 $$\rho_{nf} \left( {\frac{{\partial \mathop V\limits^{ \to } }}{\partial t} + \left( {\mathop V\limits^{ \to } .\nabla } \right)\mathop V\limits^{ \to } } \right) = \nabla .\tau - \mathop J\limits^{ \to } \times \mathop B\limits^{ \to }$$

3 $$\left( {\rho C_{p} } \right)_{nf} \left( {\frac{\partial T}{{\partial t}} + \left( {\vec{V} \cdot \nabla } \right)T} \right) = K_{nf} \nabla^{2} T + tr\left( {\tau .L} \right) + \frac{{\vec{J}^{2} }}{{\sigma_{nf} }}$$

4 $$\frac{\partial C}{{\partial t}} + \left( {\mathop V\limits^{ \to } .\nabla } \right)C = D\nabla^{2} C - Kr^{2} \left( {C - C_{\infty } } \right)\left( {\frac{T}{{T_{\infty } }}} \right)^{m} \exp \left( {\frac{ - Ea}{{kT}}} \right)$$

where

5 $$\left. {\tau = \mu A,\,\,A = \nabla \mathop V\limits^{ \to } + \left( {\nabla \mathop V\limits^{ \to } } \right)^{t} ,\,\,L = \nabla \mathop V\limits^{ \to } \,\,\,} \right\}$$

After implementing the boundary layer restrictions, flow governing model for steady flow caused by the stretched cylinder having porous walls in presence of Darcy–Forchheimer and thermal radiation becomes^5,35,36^

6 $$\frac{\partial u}{{\partial x}} + \frac{v}{r} + \frac{\partial v}{{\partial r}} = 0,$$

7 $$u\frac{\partial u}{{\partial x}} + v\frac{\partial u}{{\partial r}} = v_{nf} \left( {\frac{{\partial^{2} u}}{{\partial r^{2} }} + \frac{1}{r}\frac{\partial u}{{\partial r}} - \frac{u}{{K_{1} }}} \right) - \frac{{\sigma_{nf} B_{0}^{2} u}}{{\rho_{nf} }} - F_{e} u^{2} ,$$

8 $$u\tfrac{\partial T}{{\partial x}} + v\tfrac{\partial T}{{\partial r}} = \alpha_{nf} \left( {\tfrac{{\partial^{2} T}}{{\partial r^{2} }} + \tfrac{1}{r}\tfrac{\partial T}{{\partial r}}} \right) + \tfrac{{\sigma_{nf} B_{o}^{2} }}{{\left( {\rho C_{P} } \right)_{nf} }}u^{2} + \tfrac{1}{{\left( {\rho C_{P} } \right)_{nf} }}\tfrac{{16\sigma^{ * } T_{\infty }^{3} }}{{3k^{ * } }}\tfrac{{\partial^{2} T}}{{\partial r^{2} }} + \tfrac{{\mu_{nf} }}{{\left( {\rho C_{p} } \right)_{nf} }}\left( {\tfrac{\partial u}{{\partial r}}} \right)^{2} ,$$

9 $$u\frac{\partial C}{{\partial x}} + v\frac{\partial C}{{\partial r}} = D\left( {\frac{{\partial^{2} C}}{{\partial r^{2} }} + \frac{1}{r}\frac{\partial C}{{\partial r}}} \right) - Kr^{2} \left( {C - C_{\infty } } \right)\left( {\frac{T}{{T_{\infty } }}} \right)^{m} \exp \left( {\frac{ - Ea}{{kT}}} \right),$$

with

10 $$\left. \begin{gathered} u = u_{w} = \tfrac{{u_{0} x}}{l},\;V = 0,\;T = T_{w} ,\;C = C_{w} , \, \;at\;r = R, \hfill \\ u \to 0,\;T \to T_{\infty } ,\;C \to C_{\infty } ,\;as\;r \to \infty . \hfill \\ \end{gathered} \right\}$$

Considering

11 $$\left. \begin{gathered} u = \tfrac{{u_{0} x}}{l}f^{{^{\prime } }} (\eta ),\;v = - \tfrac{R}{r}\sqrt {\tfrac{{u_{0} \nu_{f} }}{l}} f(\eta ),\;\eta = \sqrt {\tfrac{{u_{0} }}{{l\nu_{f} }}} \tfrac{{r^{2} - R^{2} }}{2R}, \hfill \\ \theta (\eta ) = \tfrac{{T - T_{\infty } }}{{T_{w} - T_{\infty } }},\;\varphi (\eta ) = \tfrac{{C - C_{\infty } }}{{C_{w} - C_{\infty } }}. \hfill \\ \end{gathered} \right\}$$

The characteristics of CNTs for spherical nanomaterials can be written as^35,36^

12 $$\left. \begin{gathered} \mu_{nf} = \tfrac{{\mu_{f} }}{{\left( {1 - \phi_{1} } \right)^{2.5} }},\;\alpha_{nf} = \tfrac{{k_{nf} }}{{\left( {\rho C_{p} } \right)_{nf} }},\;\left( {\rho C_{p} } \right)_{nf} = \left( {1 - \phi_{1} } \right)\left( {\rho C_{p} } \right)_{f} + \phi_{1} \left( {\rho C_{p} } \right)_{CNT,} \hfill \\ \rho_{nf} = \left( {1 - \phi_{1} } \right)\rho_{f} + \phi_{1} \rho_{CNT} ,\,\,\,\,\tfrac{{k_{nf} }}{{k_{f} }} = \tfrac{{\left( {1 - \phi_{1} } \right) + 2\phi_{1} \tfrac{{k_{CNT} }}{{k_{CNT} - k_{f} }}\ln \tfrac{{k_{{CNT + k_{f} }} }}{{2k_{f} }}}}{{\left( {1 - \phi_{1} } \right) + 2\phi_{1} \tfrac{{k_{f} }}{{k_{CNT} - k_{f} }}\ln \tfrac{{k_{CNT} + k_{f} }}{{2k_{f} }}}}. \hfill \\ \end{gathered} \right\}$$

In above expression $u$ and $v$ represents components of velocity in $x$ and $r$ directions respectively, $u_{w}$ stretching velocity, $\nu_{nf} \left( { = \tfrac{{\mu_{nf} }}{{\rho_{nf} }}} \right)$ kinematic viscosity, $\rho_{nf}$ density of nanofluid, $K_{1}$ surface permeability, $\sigma_{nf}$ electrical conductivity, $F_{e} \left( { = \tfrac{{C_{b} }}{{\sqrt {k_{p} } }}} \right)$ inertial coefficient, $\alpha_{nf} \left( { = \tfrac{{k_{nf} }}{{\left( {\rho C_{P} } \right)_{nf} }}} \right)$ thermal diffusivity, $\left( {C_{P} } \right)$ specific heat, $B_{0}^{2}$ intensity of magnetic field, $\sigma^{ * }$ Stefan Boltzmann constant, $\mathop J\limits^{ \to }$ electrical current, $\mu_{nf}$ effective dynamic viscosity $k^{*}$ coefficient of mean absorption, $D$ diffusion coefficient of nanoparticles, $Kr^{2}$ reaction rate, $\phi_{1}$ the solid volume fraction of nanoparticles(SWNT), $m$ fitted constant, $Ea$ coefficient of activation energy, $R$ radius of cylinder, $T$ temperature of nanofluid and $\left( {C_{\infty } ,\,\,\,C_{w} } \right)$ the ambient and surface concentrations respectively.

Using Eqs. 6 and 7, Eqns. (1–5) takes the form;

13 $$\tfrac{1}{{\left( {1 - \phi_{1} } \right)^{2.5} \left\{ {\left( {1 - \phi_{1} } \right) + \tfrac{{\rho_{CNT} }}{{\rho_{f} }}\phi_{1} } \right\}}}\left[ {\left( {1 + 2\gamma \eta } \right)f^{{^{\prime \prime \prime } }} + 2\gamma f^{{^{\prime \prime } }} - K_{p} f^{{^{\prime } }} } \right] + ff^{{^{\prime \prime } }} - Haf^{{^{\prime } }} - (1 + F_{r} )f^{{^{{\prime^{2} }} }} = 0,$$

14 $$\left. \begin{gathered} \Pr f\theta^{{^{\prime } }} + \tfrac{{\tfrac{{k_{nf} }}{{k_{f} }}}}{{\left( {1 - \phi_{1} } \right) + \tfrac{{\phi_{1} \left( {\rho C_{p} } \right)_{CNT} }}{{\left( {\rho C_{p} } \right)_{f} }}}}\left[ {\left( {1 + 2\gamma \eta } \right)\;\theta^{{^{\prime \prime } }} + 2\gamma \theta^{{^{\prime } }} } \right] + \tfrac{1}{{\left( {1 - \phi_{1} } \right) + \tfrac{{\phi_{1} \left( {\rho C_{p} } \right)_{CNT} }}{{\left( {\rho C_{p} } \right)_{f} }}}}\tfrac{4}{3}Rd\left[ {\left( {1 + 2\gamma \eta } \right)\;\theta^{{^{\prime \prime } }} + \gamma \theta^{{^{\prime } }} } \right] \hfill \\ \quad + \tfrac{1}{{\left( {1 - \phi_{1} } \right)^{2.5} \left\{ {\left( {1 - \phi_{1} } \right) + \tfrac{{\phi_{1} \left( {\rho C_{p} } \right)_{CNT} }}{{\left( {\rho C_{p} } \right)_{f} }}} \right\}}}\Pr Ec\left( {1 + 2\gamma \eta } \right)\;f^{{\prime \prime^{2} }} + \tfrac{1}{{\left( {1 - \phi_{1} } \right) + \tfrac{{\phi_{1} \rho_{CNT} }}{{\rho_{f} }}}}\Pr Ha\;Ecf^{{^{{\prime^{2} }} }} = 0, \hfill \\ \end{gathered} \right\}$$

15 $$Scf\varphi^{{^{\prime } }} + \left[ {\left( {1 + 2\gamma \eta } \right)\;\varphi^{{^{\prime \prime } }} + 2\gamma \varphi^{{^{\prime } }} } \right] - Sc\beta \left( {1 + \delta \theta } \right)^{m} Exp\left( {\tfrac{{ - E_{1} }}{1 + \delta \theta }} \right)\varphi = 0,$$

with

16 $$\left. {\begin{array}{*{20}l} {f(0) = 0,\;f\prime (0) = 1,\;\theta (0) = 1,\;\varphi (0) = 1,\;at\;r = R,} \hfill \\ {f\prime (\infty ) \to 0,\;\theta (\infty ) \to 0,\;\varphi (\infty ) \to 0,\;as\;\eta \to \infty } \hfill \\ \end{array} } \right\}$$

In above equations $\gamma$ signifies curvature parameter, $F_{r}$ inertia coefficient, $\Pr$ Prandtl number, $Ec$ Eckert number, $Rd$ radiation parameter, $Sc$ Schmidt number, $\beta$ reaction rate parameter, $\delta$ temperature difference parameter, $Ha^{2}$ Hartmann number, $E_{1}$ activation energy variable and $K_{p}$ porosity parameter.where

17 $$\left. \begin{aligned} \gamma = & \sqrt {\tfrac{l\nu f}{{u_{0} R^{2} }}} ,\,\,F_{r} = \tfrac{{C_{b} }}{\sqrt k },\,\,\Pr = \tfrac{{\left( {\mu Cp} \right)_{f} }}{{k_{f} }},\,\,\,Ha = \sqrt {\tfrac{{\sigma_{nf} B_{0}^{2} l}}{{\rho_{f} u_{0} }}} ,\,\,Ec = \tfrac{{u_{w}^{2} }}{{Cp\left( {T_{w} - T_{\infty } } \right)}},\,\, \\ Rd = & \tfrac{{4\sigma^{*} T_{\infty }^{3} }}{{k^{ * } k_{f} }},\,\,Sc = \tfrac{\nu f}{D},\,\,\,\beta = \tfrac{{Kr^{2} }}{{u_{0} }}l,\,\,\delta = \tfrac{{T_{w} - T_{\infty } }}{{T_{\infty } }},\,\,E_{1} = \tfrac{Ea}{{kT_{\infty } }},\,\,K_{p} = \tfrac{l\nu f}{{u_{0} K_{1} }}. \\ \end{aligned} \right\}$$

### Engineering quantities

Mathematically skin friction coefficient $\left( {Cf_{x} } \right)$, Nusselt number $\left( {Nu_{x} } \right)$ and Sherwood number $\left( {Sh_{x} } \right)$ are defined as;

18 $$\left. {Cf_{x} = \, \tfrac{{2\tau_{w} }}{{\rho u_{x}^{2} }},\;Nu_{x} = \,\,\tfrac{{xq_{w} }}{{k_{f} (T_{w} - T_{\infty } )}},\,\,\,Sh_{x} = \,\,\tfrac{{xj_{w} }}{{D(C_{w} - C_{\infty } )}},} \right\}$$

where $\tau_{w}$, $q_{w}$ and $j_{w}$ respective denotes the shear stress, heat flux and mass flux and are defined as;

19 $$\left. {\tau_{w} = \mu_{nf} \left( {\tfrac{\partial u}{{\partial r}}} \right)_{r = R} ,\;q_{w} = - k_{nf} \left( {\tfrac{\partial T}{{\partial r}}} \right)_{r = R} ,\;j_{w} = - D\left( {\tfrac{\partial C}{{\partial r}}} \right)_{r = R} } \right]_{\eta = 0} ,$$

non dimensional forms can be written as

20 $$\left. {Cf_{x} Re_{x}^{{\tfrac{1}{2}}} = \tfrac{2}{{\left( {1 - \phi_{1} } \right)^{2.5} }}f^{{^{\prime \prime } }} \left( 0 \right),\;Nu_{x} Re_{x}^{{\tfrac{ - 1}{2}}} = - \tfrac{{k_{nf} }}{{k_{f} }}\theta^{{^{\prime } }} (0),\;Sh_{x} Re_{x}^{{\tfrac{ - 1}{2}}} = - \varphi^{{^{\prime } }} (0).} \right\}$$

Here $Re_{x} \left( { = \tfrac{{u_{o} x}}{l\nu }} \right)$ is local Reynolds’s number.

## Results and discussion

Here, we implemented RKF-45 to acquire the graphical and numerical computations for the nonlinear governing differential system. The RKF-45 method is widely used in scientific and engineering applications to solve ODEs because of its ability to balance accuracy and computational efficiency. It is part of a family of adaptive step-size methods that help to ensure accurate numerical solutions while minimizing the computational cost. The results are computed taking constant vales of variables $m = \gamma = 2.0$, $Fr = Ec = R = 0.2$, $K_{p} = Ha = 0.5$, $\Pr = 40.0$, $Sc = 1.2$, $E_{1} = \beta = 1.0$, $\varphi_{1} = 0.01$ and $\delta = 0.1$ for both SWCNT and MWCNT. To conform the current numerical results the reduce heat transfer rate $\left( { - \theta{\prime} \left( 0 \right)} \right)$ are compared with previously published results in Table 1. A tremendous agreement of results is perceived from Table 1. Thermophysical feature of base fluid and CNTs are given in Table 2. Behavior of velocity, thermal and mass concentration fields against involved sundry variables is examined. Furthermore, surface drag force, local heat transfer rate and Sherwood number are computed and analyzed.

**Table 1:** Comparison of results for $- \theta^{\prime} \left( 0 \right)$.

$\Pr$	Current results	Gorla and Sidawi^37^	Khan and Pop^38^
0.20	0.1690	0.1691	0.1691
0.70	0.5347	0.5349	0.4539
2.00	0.9113	0.9114	0.9113
7.00	1.8904	1.8905	1.8954
20.00	3.3538	3.3539	3.3539
70.00	6.4621	6.4622	6.4621

**Table 2:** Thermophysical properties of Propylene glycol $\left( {C_{3} H_{8} O_{2} } \right)$ and CNTs^35^.

Physical properties	Base fluid	Nanoparticles	
	Propylene glycol $\left( {C_{3} H_{8} O_{2} } \right)$	$MWCNT$	$SWCNT$
$Cp({\text{J}}/{\text{kg}}\;{\text{K}})$	$4338$	$796$	$425$
$\rho ({\text{kg}}/{\text{m}}^{{3}} )$	$938.5$	$1600$	$2600$
$k({\text{W}}/{\text{mK}})$	$0.684$	$3000$	$6600$

### Velocity field

**Figure 2:**
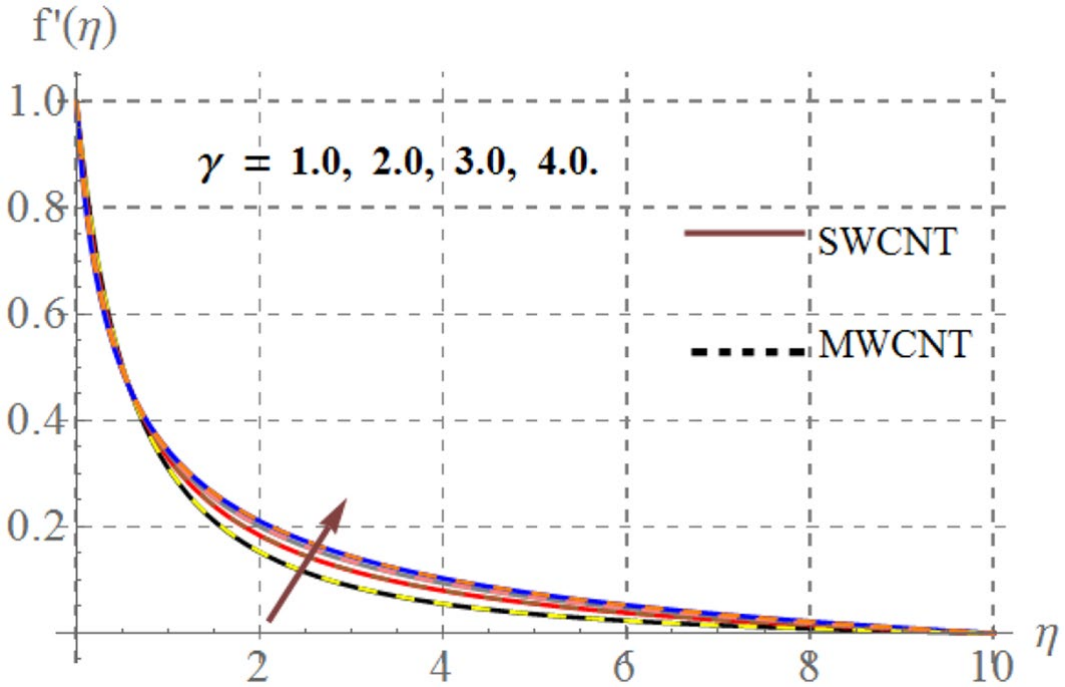
Velocity curves versus $\gamma$_._

**Figure 3:**
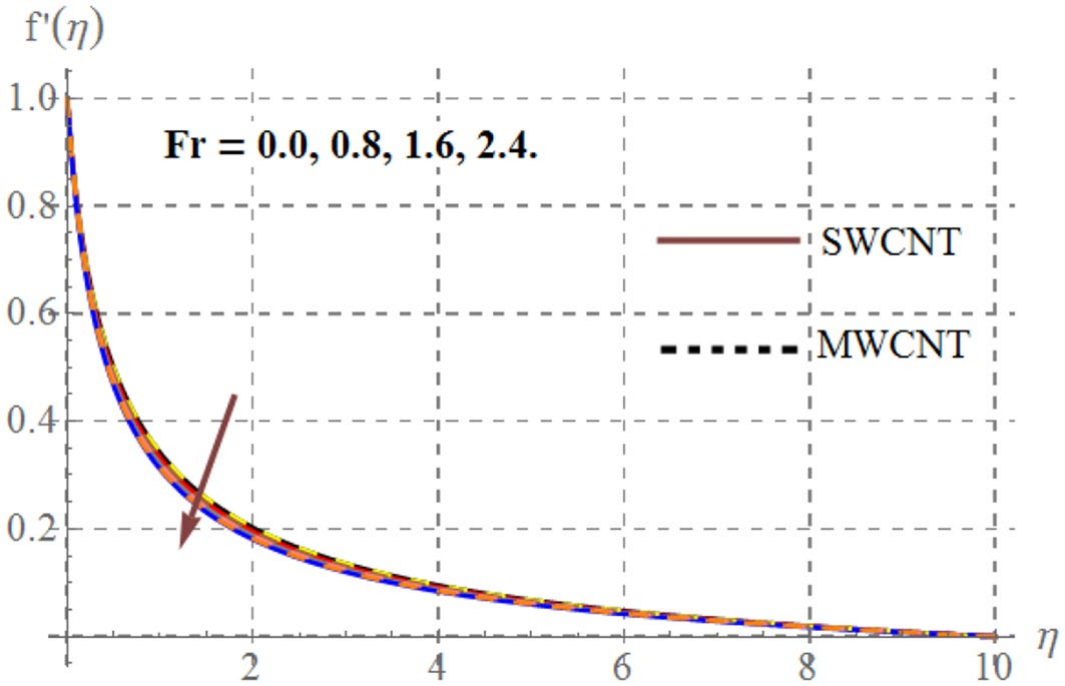
Velocity curves versus $Fr$_._

**Figure 4:**
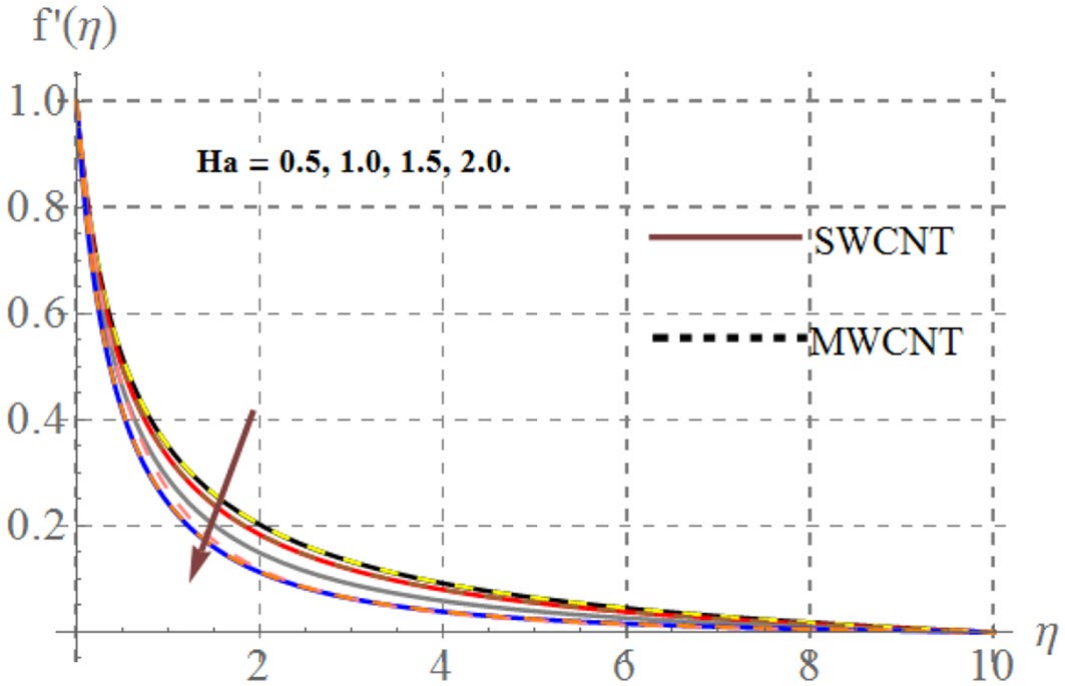
Velocity curves versus $Ha$_._

**Figure 5:**
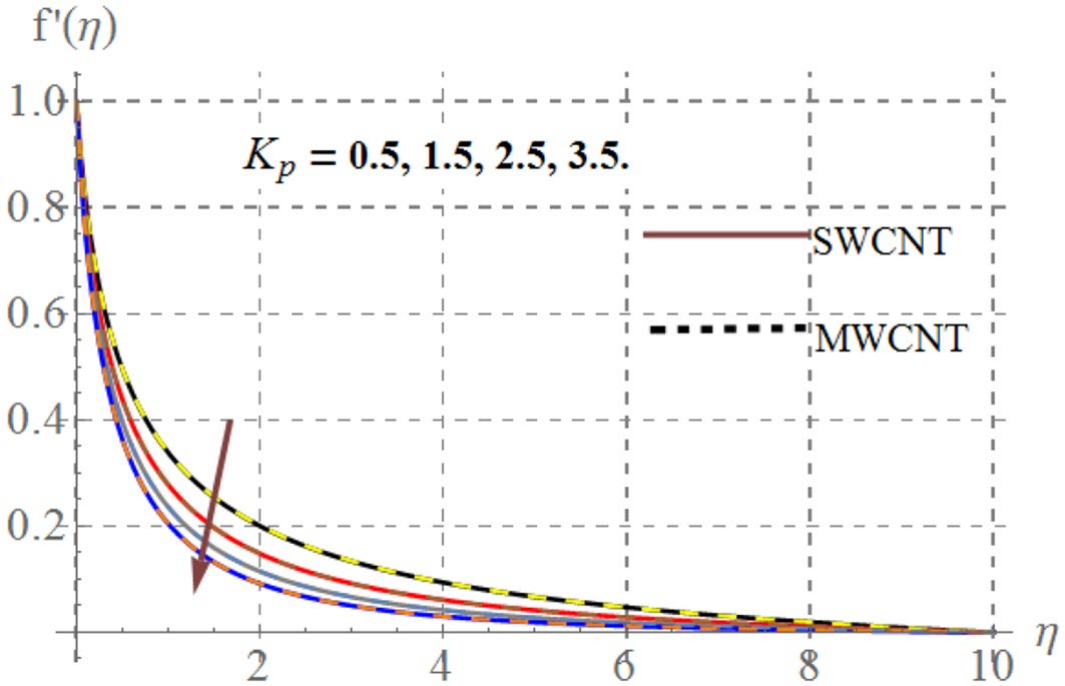
Velocity curves versus $K_{p}$.

**Figure 6:**
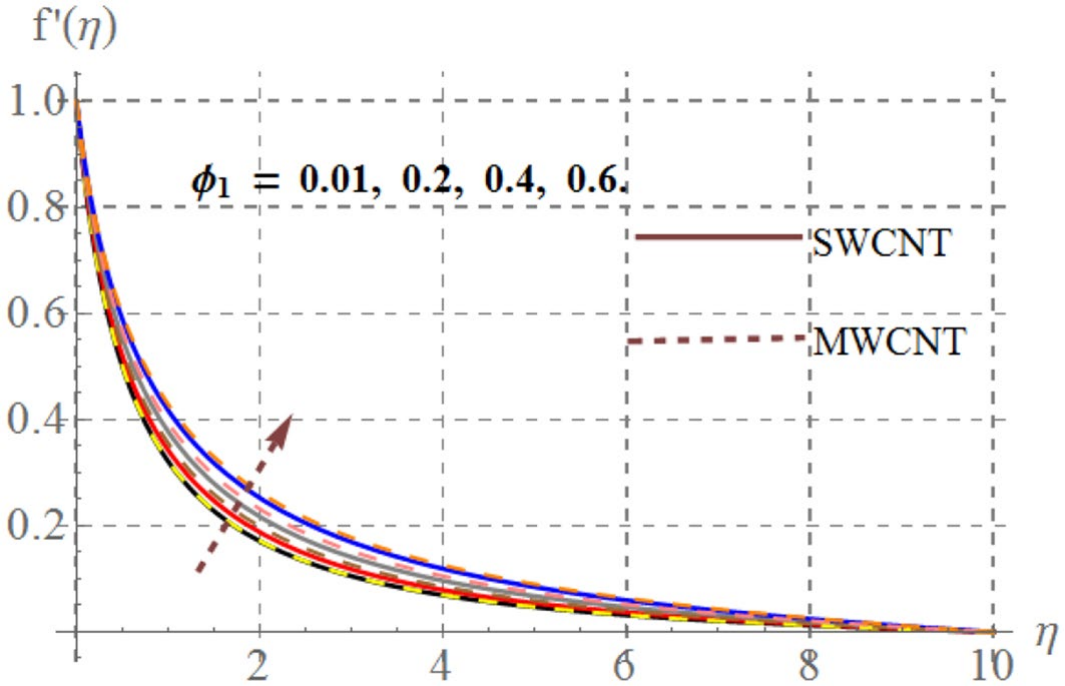
Velocity curves versus $\phi_{1}$.

Influence of diverse reflecting parameters like solid volume fraction $\left( {\phi_{1} } \right)$, curvature parameter $\left( \gamma \right)$, inertia coefficient $\left( {Fr} \right)$, Hartmann number $\left( {Ha} \right)$ and porosity parameter $\left( {K_{p} } \right)$ on velocity profile $\left( {f^{{^{\prime } }} (\eta )} \right)$ are illustrated in Figs. 2, 3, 4, 5 and 6. Fig. 2 displays the impression of $\gamma$ on velocity profile. Here one can notice that $f^{{^{\prime } }} (\eta )$ has increasing behavior through higher $\gamma$ for SWCNT/ MWCNT. Since higher $\gamma$ reduces the fluid contact area because there is an inverse relation between $\gamma$ and cylinder radius and thus velocity decays. Figure 3 shows that for higher $Fr$, velocity field diminished for SWCNT/ MWCNT. Physically, inertial forces accelerates via higher $Fr$_,_ which opposes the fluid flow and thus nanofluid velocity for SWCNT/ MWCNT retards. The aftermath of Hartmann number on fluid velocity is captured in Fig. 4. In fact larger $Ha$ corresponds to larger Lorentz force which declines the curves of velocity for SWCNT/ MWCNT. Figure 5 reveals the variation in $f^{{^{\prime } }} (\eta )$ for higher estimations of $K_{p}$. It can be noticed that velocity profile is decreased for higher values of $K_{p}$ for both SWCNT/ MWCNT. Since, size of pours of permeable surface enhances versus higher $K_{p}$ approximations, consequently resistance between surface and fluid increases and thus velocity decreases. Figure 6 is aggrandized due to extending values of $\varphi_{1}$ for SWCNT/ MWCNT. Here, $f^{{^{\prime } }} (\eta )$ accelerates versus rising $\varphi_{1}$.

### Temperature field

**Figure 7:**
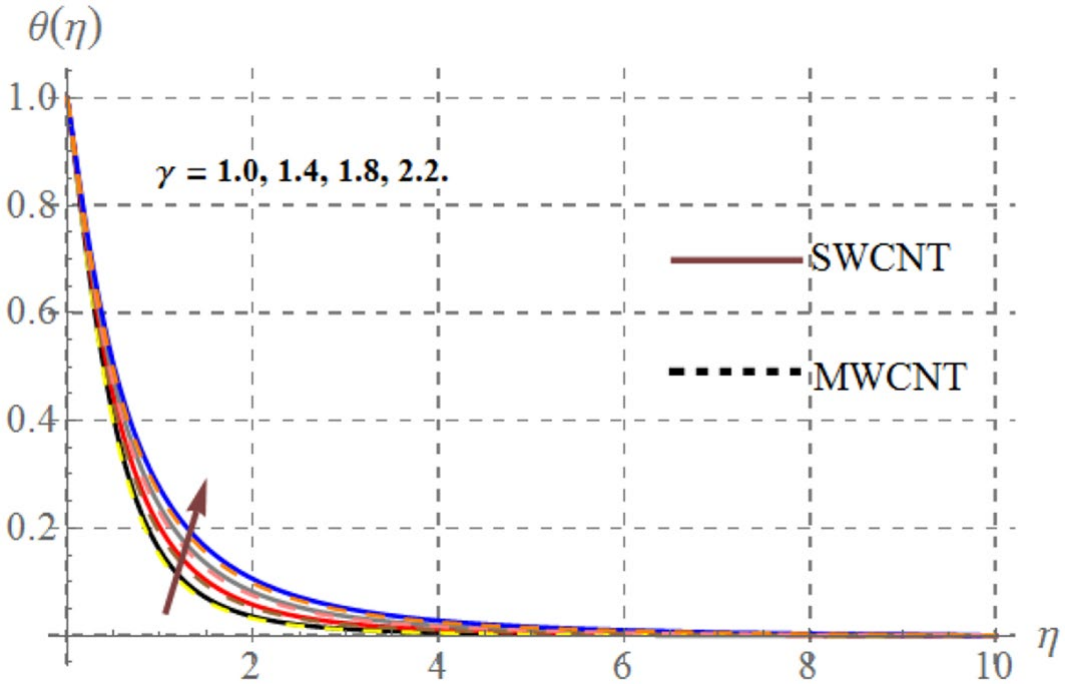
Temperature curves versus $\gamma$_._

**Figure 8:**
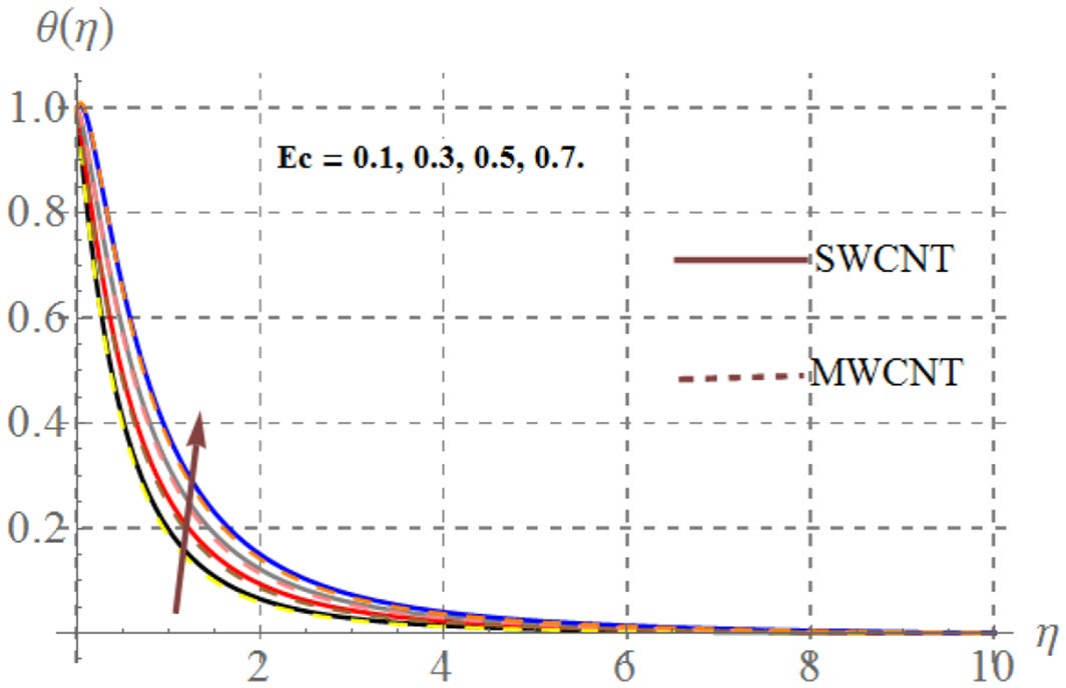
Thermal field versus $Ec$_._

**Figure 9:**
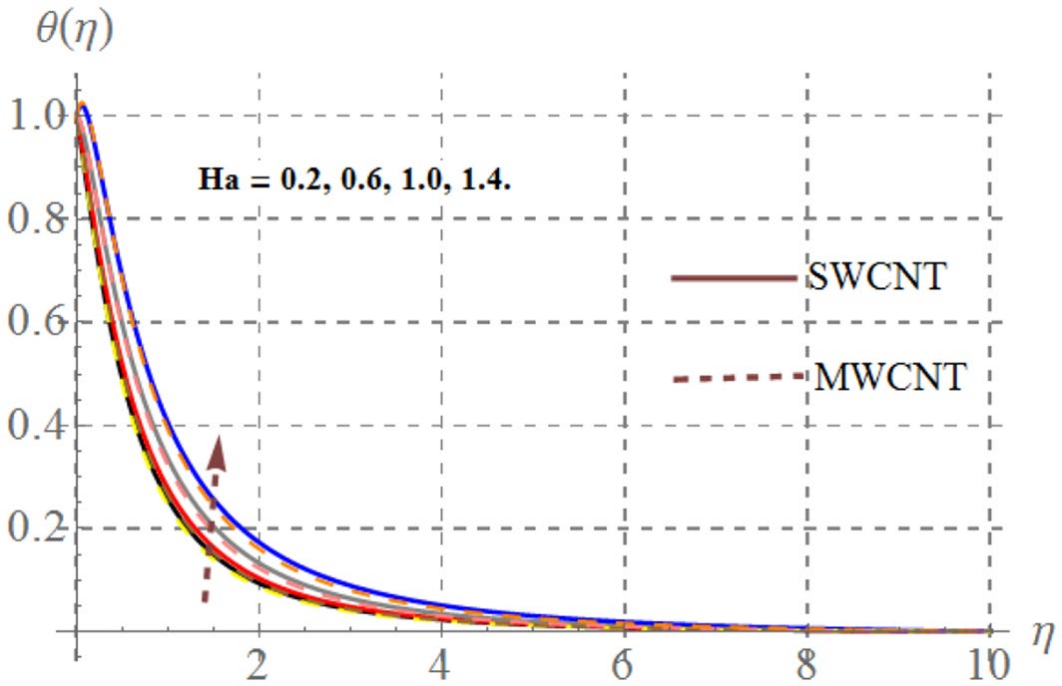
Temperature curves versus $Ha$_._

**Figure 10:**
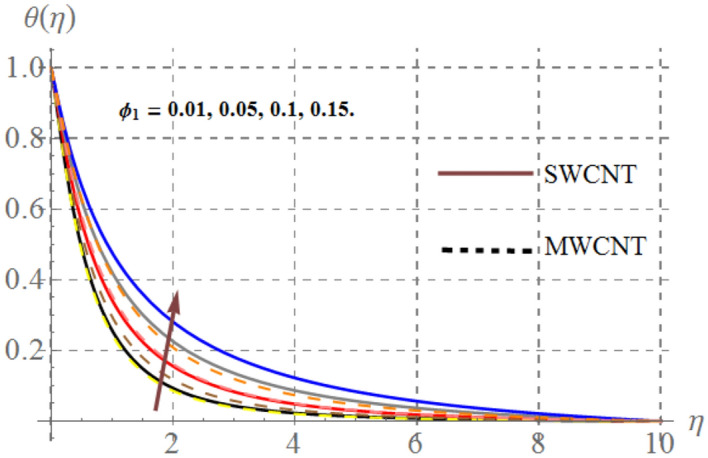
Thermal field versus $\phi_{1}$_._

**Figure 11:**
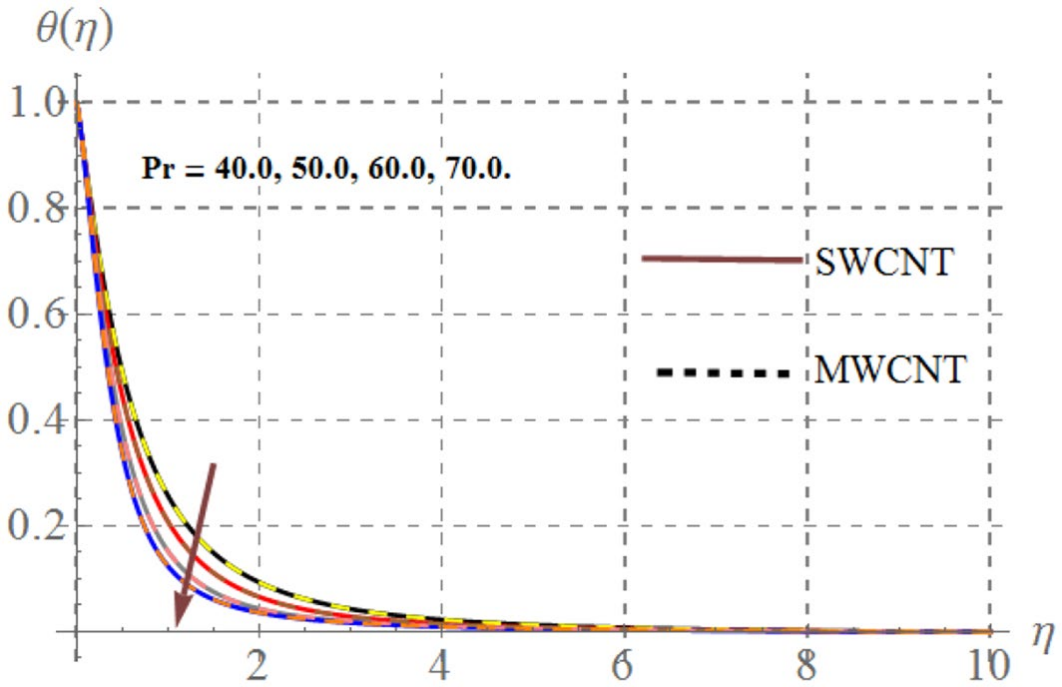
Temperature curves versus $\Pr$_._

**Figure 12:**
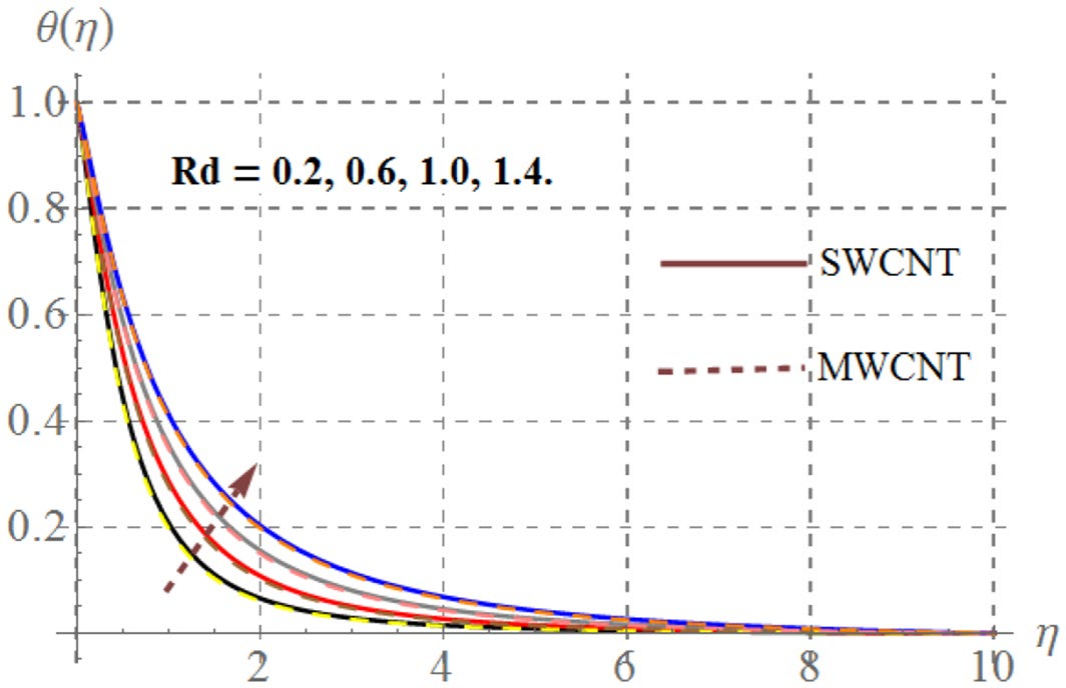
Thermal field versus $Rd$_._

Impact of curvature parameter $\left( \gamma \right)$, solid volume fraction $\left( {\phi_{1} } \right)$, Prandtl number $\left( {\Pr } \right)$, Hartmann number $\left( {Ha} \right)$, Eckert number $\left( {Ec} \right)$ and thermal radiation $\left( {Rd} \right)$ on thermal field $\left( {\theta (\eta )} \right)$ are highlighted in Figs. 7, 8, 9, 10, 11 and 12. Figure 7 demonstrates the impact of $\gamma$ on $\theta (\eta )$. Here an improvement in thermal field versus rising $\gamma$ is noticed. Since higher $\gamma$ reduces the contact area of cylinder and fluid and less amount of heat is transported from surface to the fluid thus thermal field diminished. Figure 8 captures the impact of Eckert number on $\theta (\eta )$. An augmenting change in $\theta (\eta )$ has been noted for increasing $Ec$. The accretions in Eckert number develops larger drag force between molecules of fluid. As a result more heat is generated and $\theta (\eta )$ enhances. The consequences of $Ha$ on $\theta (\eta )$ is depicted in Fig. 9. Curves of this figure indicates that temperature profile reduced as Hartmann number increased. Physically, higher $Ha$ upsurges the Lorentz resistive force and thus additional heat is added in the system, consequently thermal field boosts. The inspiration of volume fraction of nanoparticles on $\theta (\eta )$ is displayed in Fig. 10. Clearly $\theta (\eta )$ is rising function of $\phi_{1}$. Consequences of $\Pr$ on thermal field is captured in Fig. 11. Since thermal diffusivity reduces for rising $\Pr$, as a result nanofluid thermal field decays. Figure 12 is outlined to investigate the performance of $\theta (\eta )$ for higher $Rd$. It is perceived here that $Rd$ has direct relation with $\theta (\eta )$. Since higher $Rd$ provides supplementary heat to the system and thus thermal curves enhances via larger $Rd$ estimations.

### Concentration field

**Figure 13:**
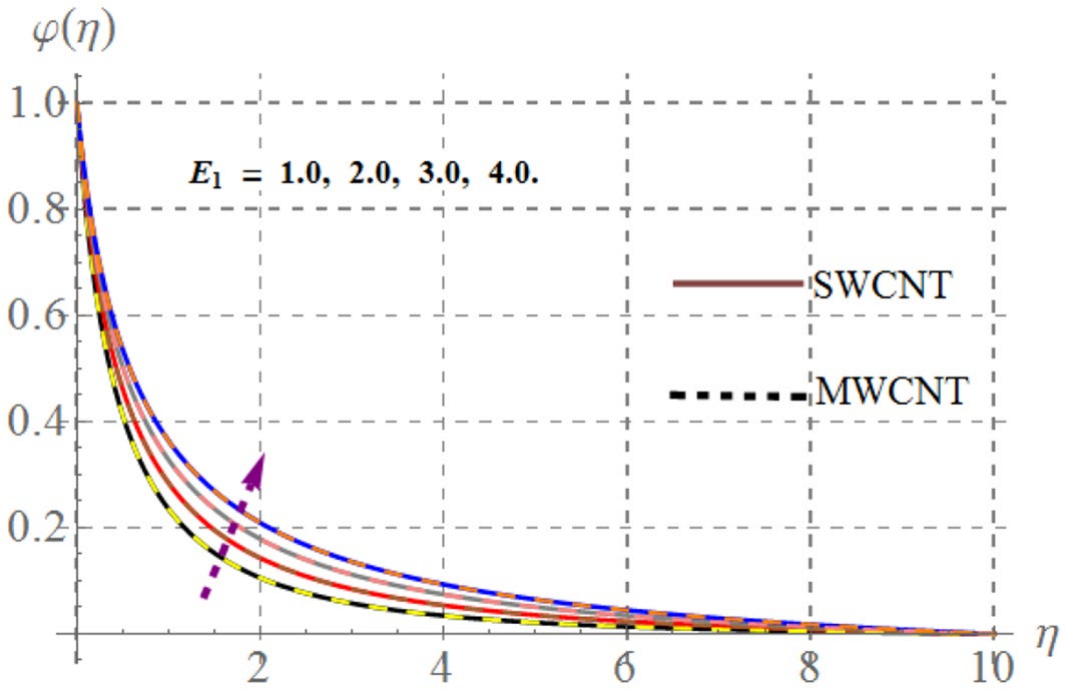
Concentration curves versus $E_{1}$.

**Figure 14:**
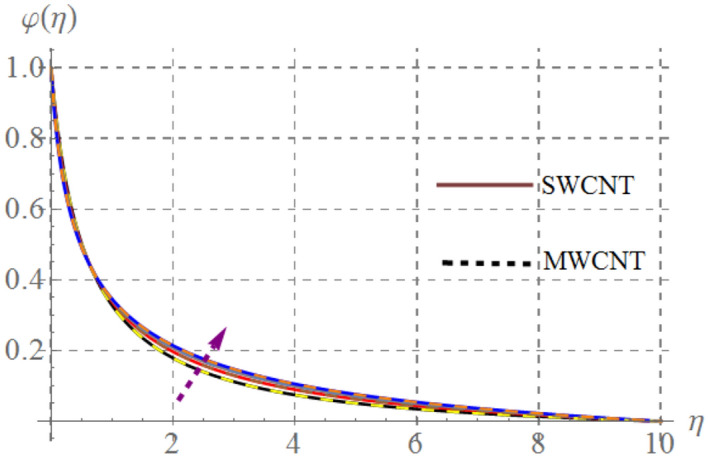
Concentration curves versus $\gamma$.

**Figure 15:**
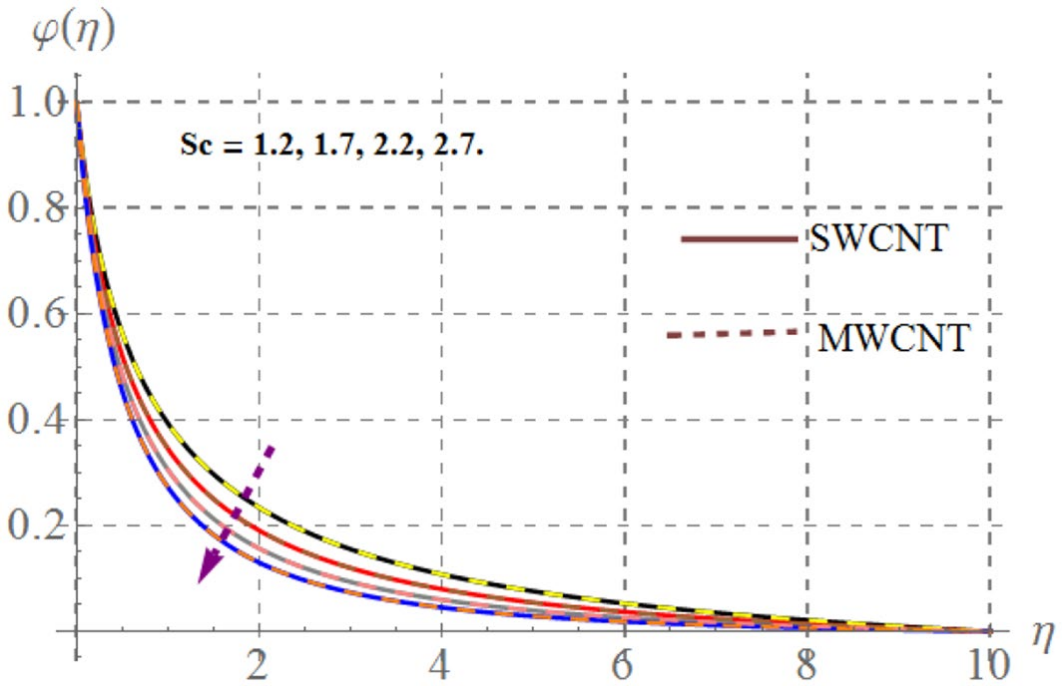
Concentration curves versus $Sc$_._

**Figure 16:**
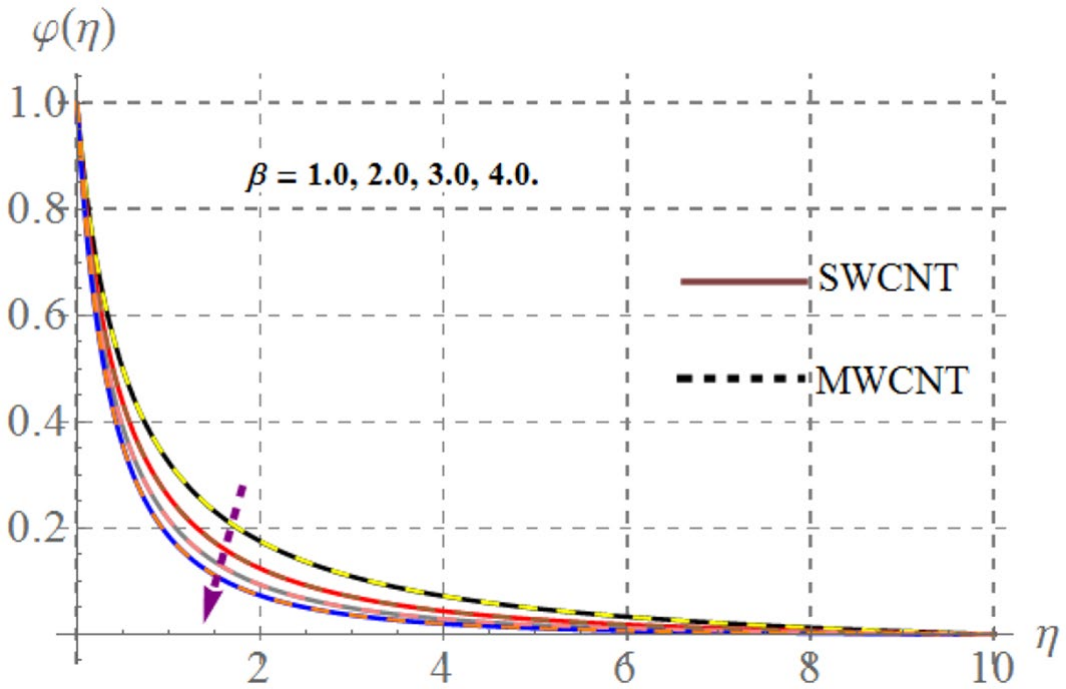
Concentration curves versus $\beta$.

Behavior of mass concentration $\left( {\varphi \left( \eta \right)} \right)$ of SWCNT/MSWCNT in Propylene glycol based fluid versus sundry variable like chemical reaction $\left( \beta \right)$, activation energy $\left( {E_{1} } \right)$, volume fraction of SWCNT/MSWCNT $\left( {\phi_{1} } \right)$, Schmidt number $\left( {Sc} \right)$ and fitted rate constant $\left( m \right)$ are examined through Figs. 13, 14, 15 and 16. Consequences of higher $E_{1}$ on $\varphi \left( \eta \right)$ is depicted in Fig. 13. Here, the intensity of $\varphi \left( \eta \right)$ escalates versus higher $\delta$. Physically, modified Arrhenius function boosts when $E_{1}$ enhances and thus $\varphi \left( \eta \right)$ increases for SWCNT/ MWCNT. Figure 14 highlights the impact of curvature on $\varphi \left( \eta \right)$, here $\varphi \left( \eta \right)$ enhances at the ambient a reverse impact is noticed at the surface of stretched cylinder. Figure 15 depicts that higher estimations of $Sc$ retards the $\varphi \left( \eta \right)$ for both SWCNT and MWCNT. Since higher $Sc$ reduces molecular mass diffusion of SWCNT and MWCNT with in the fluid. Consequently $\varphi \left( \eta \right)$ diminished. Figure 16 shows that higher approximations of chemical reaction variables reduces the $\varphi \left( \eta \right)$. In fact reactive species dissolve more rapidly versus rising $\beta$ and thus $\varphi \left( \eta \right)$ falls down for SWCNT and MWCNT.

### Physical quantities

Tables 3, 4 and 5 are prepared to examine the consequences of various parameters on skin friction coefficient $\left( {Cf_{x} Re_{x}^{{\tfrac{1}{2}}} } \right)$. Nusselt number $\left( {Nu_{x} Re_{x}^{{\tfrac{ - 1}{2}}} } \right)$ and Sherwood number for both SWCNT and MWCNT. Table 3 explores the behavior of skin friction versus higher $\phi_{1}$, $Ha$, $\gamma$, $K_{p}$ and $Fr$ SWCNT/MWCNT. It can be observe that skin friction coefficient is enhanced for higher values of $\phi_{1}$, $Ha$, $\gamma$, $K_{p}$ and $Fr$ for both SWCNT and MWCNT. Further it is noticed that magnitude of $Cf_{x} Re_{x}^{{\tfrac{1}{2}}}$ is higher for SWCNT as compared to MWCNT. Variation in $Nu_{x} Re_{x}^{{\tfrac{ - 1}{2}}}$ for rising values of $\gamma$, $Ec$, $Ha$, $\phi_{1}$, $\Pr$ and $Rd$ is listed in Table 4. Clearly Nusselt number is increasing function of $\gamma$, $\phi_{1}$ and $Rd$ while intensity of $\left( {Nu_{x} \;{\text{Re}}_{x}^{{\frac{ - 1}{2}}} } \right)$ declines for $\Pr$, $Ec$ and $Ha$ for both SWCNT/MWCNT. Table 5 describes the characteristics of $Sh_{x} \;{\text{Re}}_{x}^{{\frac{ - 1}{2}}}$ for $\gamma$, $\delta$, $\beta$, $E_{1}$, $Sc$ and $m$ in case of SWCNT/ MWCNT. Here intensity of $Sh_{x} \;{\text{Re}}_{x}^{{\frac{ - 1}{2}}}$ for SWCNT/MWCNT grows through higher $\gamma$, $\delta$, $\beta$, $Sc$ and $m$ while an opposite trend in Sherwood number is noticed for $E_{1}$.

**Table 3:** Numerical results of $Cf_{x} \,Re_{x}^{{\tfrac{1}{2}}}$ versus $\phi_{1}$, $Ha$, $\gamma$, $K_{p}$ and $Fr$.

$$\phi_{1}$$	$$Ha$$	$$\gamma$$	$$K_{p}$$	$$Fr$$	$$- Cf_{x} Re_{x}^{{\tfrac{1}{2}}}$$$$\left( {SWCNT} \right)$$	$$- Cf_{x} Re_{x}^{{\tfrac{1}{2}}}$$ $$\left( {MWCNT} \right)$$
0.01	0.2	2.0	0.3	0.2	4.00589	3.99612
0.05					4.40441	4.3547
0.1					4.97738	4.8755
	0.4				4.11675	4.1062
	0.6				4.29264	4.28089
		2.5			4.35996	4.09865
		3.0			4.70805	4.69854
			0.6		4.27748	4.26855
			0.9		4.52623	4.51797
				0.4	4.10915	4.09865
				0.6	4.20909	4.19791

**Table 4:** Numerical results of $Nu_{x} Re_{x}^{{\tfrac{ - 1}{2}}}$ versus $\gamma$, $Ec$, $Ha$, $\phi_{1}$, $\Pr$ and $Rd$.

$\gamma$	$Ec$	$Ha$	$\phi_{1}$	$\Pr$	$Rd$	$Nu_{x} Re_{x}^{{\tfrac{ - 1}{2}}}$(SWCNT)	$Nu_{x} Re_{x}^{{\tfrac{ - 1}{2}}}$(MWCNT)
2.0	$0.4$	0.2	$0.01$	20.0	0.5	1.21583	1.10583
2.5						1.23868	1.11382
$3.0$						1.26781	1.12807
	$0.8$					-0.748166	-0.801585
	$1.2$					-3.69416	-3.6627
		0.4				1.12936	1.02211
		0.6				0.992805	0.889772
			0.05			1.89231	1.33054
			0.1			2.79766	1.61657
				30.0		1.06449	0.728503
				40.0		0.861958	0.500201
					0.6	1.23868	1.11382
					1.0	1.26781	1.12807

**Table 5:** Numerical results of $Sh_{x} \;{\text{Re}}_{x}^{{\frac{ - 1}{2}}}$ versus $\gamma$, $\delta$, $\beta$, $E_{1}$, $Sc$ and $m$.

$\gamma$	$\delta$	$\beta$	$E_{1}$	$Sc$	$m$	$Sh_{x} \;{\text{Re}}_{x}^{{\frac{ - 1}{2}}}$(SWCNT)	$Sh_{x} \;{\text{Re}}_{x}^{{\frac{ - 1}{2}}}$(MWCNT)
$1.0$	0.1	$1.0$	$1.0$	1.2	2.0	1.73839	1.73817
$2.0$						1.91695	1.91685
$3.0$						2.09215	2.09213
	0.5					2.00999	1.96267
	1.0					2.23734	2.32273
		$2.0$				2.00999	2.00951
		$3.0$				2.23734	2.2367
			$2.0$			1.53859	1.53859
			$3.0$			1.45136	1.45153
				1.7		1.93593	1.9357
				2.2		2.11407	2.11383
					4.0	1.77485	1.55435
					6.0	1.81623	1.42166

## Conclusions

We have investigated the characteristics of boundary layer flow of nanofluid over stretching cylinder by using SWCNT and MWCNT as nanoparticles Propylene glycol $\left( {{\text{C}}_{{3}} {\text{H}}_{{8}} {\text{O}}_{{2}} } \right)$ is taken as based fluid. Flow governing model is developed in manifestation of Darcy–Forchheimer, permeability of surface, dissipation, thermal radiation and Arrhenius kinetics. RKF-45 technique in Mathematica package is implemented to acquire the results. Accuracy of computed results is ensured through Table 1. The main findings of current study are presented below;

- Velocity profile decays through $Fr$, $Ha$ and $K_{p}$ for SWCNT/MWCNT.
- Thermal field is increasing function of $\gamma$, $Ec$, $Ha$, $\phi_{1}$ and $Rd$ for SWCNT/MWCNT.
- Mass concentration of SWCNT/MWCNT boosts via higher $E_{1}$ and $\gamma$ whereas diminished for rising $\beta$ and $Sc.$
- Skin friction coefficient is enhanced for higher values of $\phi_{1}$, $Ha$, $\gamma$, $K_{p}$ and $Fr$ for both SWCNT and MWCNT.
- Intensity of $Sh_{x} \;{\text{Re}}_{x}^{{\frac{ - 1}{2}}}$ for SWCNT/MWCNT grows through higher $\gamma$, $\delta$, $\beta$, $Sc$ and $m$ while an opposite trend in Sherwood number is noticed for $E_{1}$.
- Magnitude of Nusselt number increases through $\gamma$, $\phi_{1}$, $\Pr$ and $Rd$ while it decays for $Ec$ and $Ha$.

### Future research directions

This work can be extended in numerous dimensions i.e., Archimedes optimization algorithm could be implemented for solution of governing equations, it could be interesting to develop mathematical models for different geometries like curved surfaces and 3-D flows. Implementation of improved Fourier and Fick’s laws for heat and mass transport could be interest of researchers.

## Data Availability

All data generated or analyzed during this study are included in this published article.

## References

[1] Iijima S (1991). Helical microtubules of graphitic carbon. Nature.

[2] Suhr J, Koratkar N, Keblinski P, Ajayan P (2005). Viscoelasticity in carbon nanotube composites. Nat. Mater..

[3] Tu JP, Yang YZ, Wang LY, Ma XC, Zhang XB (2001). Tribological properties of carbon-nanotube-reinforced copper composites. Tribol. Lett..

[4] Xue Q (2005). Model for thermal conductivity of carbon nanotube based composites. Phys. B Condens. Matter..

[5] Ramesh GK, Madhukesh JK (2021). Activation energy process in hybrid CNTs and induced magnetic slip flow with heat source/sink. Chin. J. Phys..

[6] Hayat T, Haider F, Muhammad T, Alsaedi A (2017). Three-dimensional rotating flow of carbon nanotubes with Darcy–Forchheimer porous medium. PLoS ONE..

[7] Mahesh R, Mahabaleshwar US, Sofos F (2023). Influence of carbon nanotube suspensions on Casson fluid flow over a permeable shrinking membrane: an analytical approach. Sci. Rep..

[8] Anusha T, Mahabaleshwar US, Bhattacharyya S (2023). An impact of MHD and radiation on flow of Jeffrey fluid with carbon nanotubes over a stretching/shrinking sheet with Navier’s slip. J. Therm. Anal. Calorim..

[9] Sudarsana Reddy P, Jyothi K, Suryanarayana RM (2018). Flow and heat transfer analysis of carbon nanotubes-based Maxwell nanofluid flow driven by rotating stretchable disks with thermal radiation. J. Braz. Soc. Mech. Sci. Eng..

[10] Raja MAZ, Farooq U, Chaudhary NI, Wazwaz AM (2016). Stochastic numerical solver for nanofluidic problems containing multi-walled carbon nanotubes. Appl. Soft Comput..

[11] Raja MAZ, Sabati M, Parveen N, Awais M, Awan SE, Chaudhary NI (2021). Integrated intelligent computing application for effectiveness of Au nanoparticles coated over MWCNTs with velocity slip in curved channel peristaltic flow. Sci. Rep..

[12] Alkuhayli NAM (2023). Magnetohydrodynamic flow of copper-water nanofluid over a rotating rigid disk with Ohmic heating and hall effects. J. Magn. Magn. Mater..

[13] Alzabut J, Nadeem S, Noor S, Eldin SM (2023). Numerical analysis of Magnetohydrodynamic convection heat flow in an enclosure. Results Phys..

[14] Sadighi S, Afshar H, Jabbari M, Ahmadi Danesh Ashtiani H (2023). Heat and mass transfer for MHD nanofluid flow on a porous stretching sheet with prescribed boundary conditions. Case Stud. Therm. Eng..

[15] Jakeer S, Reddy SRR, Easwaramoorthy SV, Basha HT, Cho J (2023). Exploring the influence of induced magnetic fields and double-diffusive convection on carreau nanofluid flow through diverse geometries: a comparative study using numerical and ANN approaches. Mathematics.

[16] Rasool G, Zhang T, Chamkha AJ, Shafiq A, Tlili I, Shahzadi G (2020). Entropy generation and consequences of binary chemical reaction on MHD Darcy–Forchheimer Williamson nanofluid flow over non-linearly stretching surface. Entropy.

[17] Hayat T, Shafiq A, Alsaedi A (2015). MHD axisymmetric flow of third grade fluid by a stretching cylinder. Alex. Eng. J..

[18] Shafiq A, Çolak AB, Sindhu TN (2022). Significance of bioconvective flow of MHD thixotropic nanofluid passing through a vertical surface by machine learning algorithm. Chin. J. Phys..

[19] Awais M, Salahuddin T (2023). Radiative magnetodydrodynamic cross fluid thermophysical model passing on parabola surface with activation energy. Ain Shams Eng. J..

[20] Forchheimer P (1901). Wasserbewegung durch boden. Z Ver Deutsch Ing..

[21] Muskat M. The flow of homogeneous fluids through porous media: Ann Arbor. Michigan, JW Edwards 763 (1946).

[22] Seddeek MA (2006). Influence of viscous dissipation and thermophoresis on Darcy–Forchheimer mixed convection in a fluid saturated porous media. J Colloid Interface Sci..

[23] Vishnu Ganesh N, Abdul Hakeem AK, Ganga B (2018). Darcy–Forchheimer flow of hydromagnetic nanofluid over a stretching/shrinking sheet in a thermally stratified porous medium with second order slip, viscous and Ohmic dissipations effects. Ain Shams Eng. J..

[24] Jawad M, Hameed MK, Nisar KS, Majeed AH (2023). Darcy–Forchheimer flow of maxwell nanofluid flow over a porous stretching sheet with Arrhenius activation energy and nield boundary conditions. Case Stud. Therm. Eng..

[25] Ullah MZ, Serra-Capizzano S, Baleanu D (2020). A numerical simulation for Darcy–forchheimer flow of nanofluid by a rotating disk with partial slip effects. Front. Phys..

[26] Çolak AB, Shafiq A, Sindhu TN (2022). Modeling of Darcy–Forchheimer bioconvective Powell Eyring nanofluid with artificial neural network. Chin. J. Phys..

[27] Raja MAZ, Khan Z, Zuhra S, Chaudhary NI, Khan WU, He Y (2021). Cattaneo-christov heat flux model of 3D hall current involving biconvection nanofluidic flow with Darcy–Forchheimer law effect: Backpropagation neural networks approach. Case Stud. Therm. Eng..

[28] Awais M, Salahuddin T, Muhammad S (2023). Effects of viscous dissipation and activation energy for the MHD Eyring-powell fluid flow with Darcy–Forchheimer and variable fluid properties. Ain Shams Eng. J..

[29] Upreti H, Pandey AK, Kumar M, Makinde OD (2022). Darcy–Forchheimer flow of CNTs-H_2_O nanofluid over a porous stretchable surface with Xue model. Int. J. Mod. Phys. B..

[30] Essam ME, Abedel-AaL EM (2023). Darcy–Forchheimer flow of a nanofluid over a porous plate with thermal radiation and brownian motion. J. Nanofluids..

[31] Rahman M, Haq F, Darab PC, Sallah M, Abdelmohsen SAM, Fadhl BM (2023). Mixed convection and activation energy impacts on MHD bioconvective flow of nanofluid with irreversibility assessment. Heliyon.

[32] Sahu SK, Rout S, Shaw S, Dash N, Thatoi DN, Nayak MK (2022). Hydrothermal stagnation point flow of Carreau nanofluid over a moving thin needle with non-linear Navier's slip and cubic autocatalytic chemical reactions in Darcy–Forchheimer medium. J. Indian Chem. Soc..

[33] Rasool G, Wakif A, Wang X, Alshehri A, Saeed AM (2023). Falkner-Skan aspects of a radiating (50% ethylene glycol + 50% water)-based hybrid nanofluid when Joule heating as well as Darcy–Forchheimer and Lorentz forces affect significantly. Propuls. Power Res..

[34] Ijaz Khan M, Hayat T, Shah F, Mujeeb Ur R, Haq F (2019). Physical aspects of CNTs and induced magnetic flux in stagnation point flow with quartic chemical reaction. Int. J. Heat Mass Transf..

[35] Shaiq S, Maraj EN (2019). Role of the induced magnetic field on dispersed CNTs in propylene glycol transportation toward a curved surface. Arab. J. Sci. Eng..

[36] Prashar P, Ojjela O (2022). Numerical investigation of ZnO–MWCNTs/ethylene glycol hybrid nanofluid flow with activation energy. Indian J. Phy..

[37] Reddy Gorla RS, Sidawi I (1994). Free convection on a vertical stretching surface with suction and blowing. Appl. Sci. Res..

[38] Khan WA, Pop I (2010). Boundary-layer flow of a nanofluid past a stretching sheet. Int. J. Heat Mass Transf..

